# Role of cognitive reserve in cognitive variability in euthymic individuals with bipolar disorder: cross-sectional cluster analysis

**DOI:** 10.1192/bjo.2020.111

**Published:** 2020-10-30

**Authors:** Dimosthenis Tsapekos, Rebecca Strawbridge, Tim Mantingh, Matteo Cella, Til Wykes, Allan H. Young

**Affiliations:** Department of Psychological Medicine, Institute of Psychiatry, Psychology and Neuroscience, King's College London, UK; Department of Psychological Medicine, Institute of Psychiatry, Psychology and Neuroscience, King's College London, UK; Department of Psychological Medicine, Institute of Psychiatry, Psychology and Neuroscience, King's College London, UK; Department of Psychology, Institute of Psychiatry, Psychology and Neuroscience, King's College London; and South London & Maudsley NHS Foundation Trust, Maudsley Hospital, London, UK; Department of Psychology, Institute of Psychiatry, Psychology and Neuroscience, King's College London; and South London & Maudsley NHS Foundation Trust, Maudsley Hospital, London, UK; Department of Psychological Medicine, Institute of Psychiatry, Psychology and Neuroscience, King's College London; and South London & Maudsley NHS Foundation Trust, Maudsley Hospital, London, UK

**Keywords:** Bipolar disorder, clustering, cognitive profiles, cognitive reserve, cognitive remediation

## Abstract

**Background:**

People with bipolar disorder have moderate cognitive difficulties that tend to be more pronounced during mood episodes but persist after clinical remission and affect recovery. Recent evidence suggests heterogeneity in these difficulties, but the factors underlying cognitive heterogeneity are unclear.

**Aims:**

To examine whether distinct cognitive profiles can be identified in a sample of euthymic individuals with bipolar disorder and examine potential differences between subgroups.

**Method:**

Cognitive performance was assessed across four domains (i.e. processing speed, verbal learning/memory, working memory, executive functioning) in 80 participants. We conducted a hierarchical cluster analysis and a discriminant function analysis to identify cognitive profiles and considered differences in cognitive reserve, estimated cognitive decline from premorbid cognitive functioning, and clinical characteristics among subgroups.

**Results:**

Four discrete cognitive profiles were identified: cognitively intact (*n* = 25; 31.3%); selective deficits in verbal learning and memory (*n* = 15; 18.8%); intermediate deficits across all cognitive domains (*n* = 30; 37.5%); and severe deficits across all domains (*n* = 10; 12.5%). Cognitive decline after illness onset was greater for the intermediate and severe subgroups. Cognitive reserve scores were increasingly lower for subgroups with greater impairments. A smaller proportion of cognitively intact participants were using antipsychotic medications compared with all other subgroups.

**Conclusions:**

Our findings suggest that individuals with cognitively impaired profiles demonstrate more cognitive decline after illness onset. Cognitive reserve may be one of the factors underlying cognitive variability across people with bipolar disorder. Patients in the intermediate and severe subgroups may be in greater need of interventions targeting cognitive difficulties.

Bipolar disorder is a mental health condition characterised by recurrent episodes of depression and (hypo)mania. Recent findings suggest that people with bipolar disorder experience moderate cognitive impairments, 0.5–1 standard deviation (s.d.) below the normative mean, which are more pronounced during mood episodes but persist after the symptoms remit, during periods of euthymia.^1,2^ Deficits appear across multiple domains, including processing speed, verbal memory and executive functioning.^3^ Considerable evidence suggests that these impairments affect daily life functioning, possibly independent of mood symptoms.^4^ Findings indicate that cognitive difficulties are present in a proportion of patients with bipolar disorder,^5,6^ and more recently this heterogeneity has been characterised through the identification of different profiles of cognitive difficulties.

The evidence suggests that there are three or four discrete and coherent profiles, one cognitively intact and comparable to the general population, plus one or two subgroups presenting with selective moderate impairments, and a globally impaired subgroup with severe impairments across cognitive domains.^7^ Similar findings have been reported from studies with cross-diagnostic samples involving people with different diagnoses across the psychosis spectrum.^8,9^

There is also a limited understanding of the factors underlying cognitive heterogeneity in bipolar disorder. An aspect of cognitive heterogeneity remaining unexplored is whether cognitive clusters represent different degrees of cognitive decline following illness onset and the extent of this putative decline across subgroups. A recent study with a cross-diagnostic sample defined cognitive decline as the discrepancy between current cognitive performance and premorbid IQ and reported evidence for a cluster characterised by a large cognitive decline, but limited changes for the other subgroups.^10^

Cognitive reserve might be a factor underlying this difference in cognitive decline between clusters.^11^ It reflects resilience to brain pathology by minimising its effect on behavioural outcomes, such as symptom manifestation or cognitive dysfunction.^12,13^ Several proxy measures of cognitive reserve have been suggested, including years of education, occupational attainment and measures of vocabulary knowledge or reading abilities.^14^ The concept of reserve has been extensively explored in neurological and psychiatric disorders such as dementia, multiple sclerosis and schizophrenia, representing individual differences in the capacity to compensate for age- and illness-related cognitive decline in the presence of neuropathology.^14–16^ Previous research in bipolar disorder suggests an association of cognitive reserve with cognitive performance,^17^ but this has not been explored in the context of cognitive heterogeneity, particularly whether and to what extent cognitive differences between putative subgroups can be explained as a function of cognitive reserve.

Illness-history variables, such as the type and the number of previous mood episodes, have been investigated as putative underlying factors of cognitive heterogeneity. However, previous studies have reported inconsistent findings on the association of clinical characteristics and medication use with the profile of cognitive impairment.^7^ The inclusion of participants partially remitted or with subsyndromal symptoms may have resulted in these mixed findings between studies. Hence, it remains unclear whether cognitive performance across cognitive clusters is affected by patient differences in illness-related characteristics.

Here, we examine whether discrete cognitive profiles can be identified within a cohort of euthymic patients with bipolar disorder and test the hypothesis that cognitive reserve will differ between subgroups. We anticipate replicating the findings of previous cluster-analytic studies reporting on euthymic participants^18,19^ and we use independent cognitive measures to internally validate the identified clusters. Cognitive reserve and estimated postmorbid cognitive decline are examined as factors underlying potential differences in the cognitive course of putative subgroups. Differences in clinical characteristics are also examined among clusters. Parsing cognitive heterogeneity in bipolar disorder is important for delivering targeted interventions and potentially improving outcomes. A better understanding of the underlying factors associated with cognitive clustering may also inform intervention strategies, as well as help clinical services recognise earlier or more efficiently which patients require cognitive treatment.

## Method

### Study design

This is a cross-sectional secondary analysis of baseline data from the Cognitive Remediation in Bipolar (CRiB) study, a feasibility trial comparing cognitive remediation with treatment as usual in people with bipolar disorder.^20,21^ Written informed consent was obtained from all participants prior to inclusion. Baseline assessments were undertaken before random allocation to the treatment or the control group. The authors assert that all procedures contributing to this work comply with the ethical standards of the relevant national and institutional committees on human experimentation and with the Helsinki Declaration of 1975, as revised in 2008. All procedures involving human patients were approved by the City Road & Hampstead NHS Research Ethics Committee (reference 15/LO/1557).

### Participants

The sample comprised 80 out-patients with a DSM-5 diagnosis of bipolar disorder. All participants were fluent in English and aged between 18 and 65 years. Bipolar disorder subtype and eligibility were confirmed using the Mini International Neuropsychiatric Interview.^22^ Participants were free of acute mood symptoms for at least 1 month prior to inclusion. Remission at screening stage was defined using cut-off scores of ≤7 on the 17-item Hamilton Rating Scale for Depression (HRSD)^23^ and the Young Mania Rating Scale (YMRS).^24^ Participants with a diagnosed neurological disorder or personality disorder and those misusing or dependent on alcohol or illicit substances over the previous 6 months were excluded.

### Measures

#### Clinical assessment

A structured interview was used to collect information on demographic characteristics, illness-history variables and current medication use. Mood symptoms were assessed using the HRSD for depressive and the YMRS for hypomanic/manic symptoms.

#### Cognitive assessment

##### Clustering measures

Participants were administered an extensive cognitive battery in a standardised order. The battery included eight tests to be used as clustering tests: the Hotel test,^25^ Wechsler Memory Scale Fourth Edition (WMS-IV) Verbal Paired Associates immediate and delayed recall (VPA I and II),^26^ Wechsler Abbreviated Scale of Intelligence Second Edition (WASI-II) Matrix Reasoning subset,^27^ Wechsler Adult Intelligence Scale Fourth Edition (WAIS-IV) Digit Span, Digit Symbol–Coding and Symbol Search,^28^ and the F-A-S letter verbal fluency test from the Delis–Kaplan Executive Function System.^29^

##### Non-clustering measures

Two measures of general cognitive performance were used for the internal validation of the emerging cognitive subgroups.^10^ Participants were administered the Montreal Cognitive Assessment (MoCA),^30^ a compact screening instrument assessing multiple cognitive domains and giving a single score. We also used the cognitive subscale of the Functional Assessment Short Test (FAST),^31^ a clinician-rated measure examining cognitive functioning in the context of daily life situations and activities, with higher scores indicating greater difficulties.

##### IQ measures

Two intelligence measures were administered: an estimate of premorbid IQ, the Test of Premorbid Function (TOPF),^32^ and an estimate of verbal IQ, the Vocabulary subtest of the WASI-II.^27^ Both represent cognitive reserve as measures of crystallised intelligence and have been shown to be resistant to the effects of ageing or illness-related decline.^33^

### Statistical analysis

Despite a lack of consensus on the required sample size to achieve adequate statistical power in cluster analysis, a minimum sample size of 2*^k^* (where *k* is the number of clustering variables) has been suggested, although the ideal sample size would be five times this number.^34^ On the basis of this recommendation and our sample of 80 participants, we considered four clustering variables in this study.

All statistical analyses were conducted using the Statistical Package for Social Sciences (SPSS), version 25 for Windows, with a statistical significance of *P* < 0.05 (two-tailed) for all tests. The distributions were checked for normality across all measures using the Shapiro–Wilk test and log transformation was applied to conform non-normally distributed variables. Descriptive statistics were computed for all variables.

#### Cognitive domains for clustering

Raw scores of cognitive tests were transformed to demographically corrected standardised scores (*z-*scores; mean 0, s.d. = 1) based on the normative data for each test as provided by manuals. The Hotel test *z*-scores were inverted to be consistent with the direction of other measures, since for this test higher scores represent poorer performance. We computed *z*-scores for four cognitive domains: processing speed using the average scores of the Digit Symbol–Coding and the Symbol Search; working memory with the Digit Span forward, backward and sequencing scores; verbal learning and memory using the average scores of the VPA I and VPA II; and executive functioning using the average scores of the Hotel test, Matrix Reasoning and F-A-S letter verbal fluency test. A composite score of current global cognition was computed for each participant by averaging the *z*-scores of all the tests used for the clustering domains.

#### Identifying and validating cognitive clusters

The four cognitive domains were entered into a hierarchical cluster analysis to identify subgroups with homogeneous cognitive profiles according to their performance in these domains. Following Burdick et al,^11^ we used Ward's linkage as the clustering method and squared Euclidean distance to estimate similarities between cases. To validate the initial clustering and evaluate the accuracy of participant allocation across clusters, we conducted a discriminant function analysis (DFA). The optimal number of clusters was determined on the basis of visual inspection of the dendrogram and the DFA coefficients. DFA examines the predictive power of different domain scores for every subgroup identified in the hierarchical cluster analysis and determines the probability of classification into a certain subgroup for every participant on the basis of these scores.

To estimate in which domains and to what extent these subgroups are distinct, cognitive profiles were compared to each other using multivariate analysis of covariance (MANCOVA) with least significant difference (LSD) for pairwise comparisons. Age was used as a covariate since age differences were evident between subgroups. For MANCOVAs with a significant main effect, *post hoc* comparisons between subgroups were corrected for multiple testing using a false discovery rate (FDR) of 5%.

Given that the cluster analysis was applied to differentiate participants on the basis of their cognitive performance, we anticipated significant between-group differences that would internally validate the cognitive clustering of the sample. Cohen's *d* effect sizes (defined as the mean group difference divided by the pooled s.d. and corrected for unequal group sizes) were calculated for significant *post hoc* comparisons. To further validate within the sample that the emerging cognitive profiles are truly distinct and not just artefacts of the measures used to identify them, subgroups were compared on non-clustering cognitive measures (MoCA and FAST cognitive subscale) using MANCOVA with age as a covariate and correcting for multiple comparisons with a 5% FDR.

#### Estimating cognitive decline and cognitive reserve

A proxy measure of postmorbid cognitive decline was estimated for each participant as the discrepancy between the current global cognitive performance (composite score) and the estimated premorbid IQ (TOPF score), following the methodology of previous studies.^8,10^ Both the global cognition composite and the TOPF score were standardised to the normative performance of healthy controls according to the validation data.

Cognitive reserve was estimated for each participant through a composite score of three proxy measures: years of education and the two intelligence indices, the TOPF and the Vocabulary subtest of the WASI-II, according to previous recommendations.^15,16^ Using a factor analysis, a single score was derived from these three variables for each participant.^15^ This factor score accounted for 59.8% of the shared variance in the three variables and was used as a measure of cognitive reserve in all subsequent analyses. To ensure that cognitive reserve is an estimate independent of ageing and illness progression, this score was correlated to participants’ age, age at illness onset, illness duration and number of mood episodes.

Differences between subgroups in cognitive decline and cognitive reserve were examined using a MANCOVA (age as covariate) with an FDR of 5% to correct for multiple comparisons.

#### Comparisons on demographic and clinical characteristics

Subgroup comparisons were conducted to examine differences in non-cognitive variables using chi-squared (*χ*^2^) tests or analyses of variance (ANOVAs) with Games–Howell *post hoc* correction for unequal variances and small sample sizes. Identified clusters were compared on demographic characteristics, clinical history, current mood symptoms, use of medication and previous service use. To estimate the magnitude of potential differences, Cohen's *d* was calculated using the same procedure. Cohen's *w* effect sizes were calculated for categorical variables.

## Results

### Sample characteristics

Table 1 provides details on the demographic and clinical characteristics of sample. There were no missing data for any clinical variables or cognitive tests. Overall, the sample performed mildly below the normative scores in two domains: verbal learning and memory (*z* = −0.26, s.d. *=* 1.1) and executive functioning (*z* = −0.22, s.d. *=* 0.66). Even smaller differences were detected in processing speed (*z* = −0.14, s.d. *=* 0.72), working memory (*z* = −0.08, s.d. *=* 0.61) and global cognition (*z* = −0.17, s.d. *=* 0.61).

**Table 1:** Sample demographic and clinical characteristics (*N* = 80)

	Mean (s.d.)	Minimum–Maximum
Age	42.2 (12.8)	19–65
Education, years	15.9 (2.1)	11–21
Estimated premorbid IQ (TOPF)	109.2 (6.9)	93–121
Age bipolar disorder diagnosed, years	30.9 (11.7)	16–61
Diagnosis duration, years	10.8 (8.9)	0–34
Symptom duration, years	21.5 (12.7)	1–56
Duration of untreated illness, years	10.7 (9.6)	0–44
Number of depressive episodes	12.4 (14.3)	1–91
Number of (hypo)manic	8.5 (7.2)	1–32
Number of hospital admissions	2.4 (2.9)	0–14
Current euthymia, months	13.8 (24.6)	1–168
Number of current medications	2.4 (1.5)	0–7
Number of psychological therapies undertaken	1.96 (1.54)	0–8
HRSD-17 score	3.3 (2.2)	0–7
YMRS score	2.1 (2.1)	0–7

	*n*	%

Gender, female/male	57/23	71/29
Employed	37	46
Bipolar disorder type, I/II	53/27	66/34
Family history of affective disorders	43	54
History of psychosis	52	65
Current medication class		
Lithium	26	33
Anticonvulsants	47	59
Antipsychotics	59	74
Antidepressants	39	49
Anxiolytics	13	16

TOPF, Test of Premorbid Function; HRSD-17, 17-item Hamilton Rating Scale for Depression; YMRS, Young Mania Rating Scale.

### Identifying cognitive clusters

**Fig. 1:**
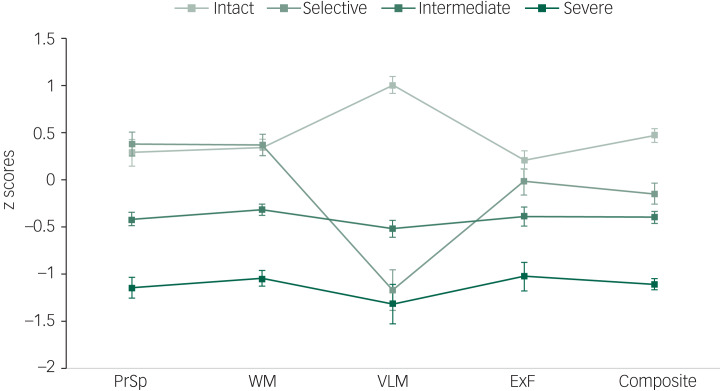
Cognitive profiles (domain mean and standard error) of the four subgroups (cognitively intact; selective deficit in verbal learning and memory; intermediate impairment across all domains; and severe global impairments). PrSp, processing speed; WM, working memory; VLM, verbal learning and memory; ExF, executive functioning; Composite, composite cognitive score.

Inspection of the dendrogram and the agglomeration coefficients suggested a four-cluster solution as the most appropriate fit for the sample: a subgroup of 25 participants (31%) remained cognitively intact, another subgroup of 15 participants (19%) had a selective deficit in verbal learning and memory, a third subgroup of 30 participants (37.5%) had intermediate impairment across all domains and a fourth subgroup of 10 participants (12.5%) had severe global impairments. The intact cluster performed slightly above the normative mean in three domains (by 0.2–0.3 s.d.) and 1 s.d. above the normative mean in verbal learning and memory. The second cluster showed a comparable performance to the intact subgroup in processing speed, working memory and executive functioning, but presented a selective impairment of 1 s.d. below the normative mean in verbal learning and memory. The intermediate cluster consistently performed 0.3–0.5 s.d. below the normative mean across all domains. The last cluster was characterised by severe impairments exceeding 1 s.d. below the norm in all domains. Figure 1 illustrates the cognitive profiles of the subgroups.

**Fig. 2:**
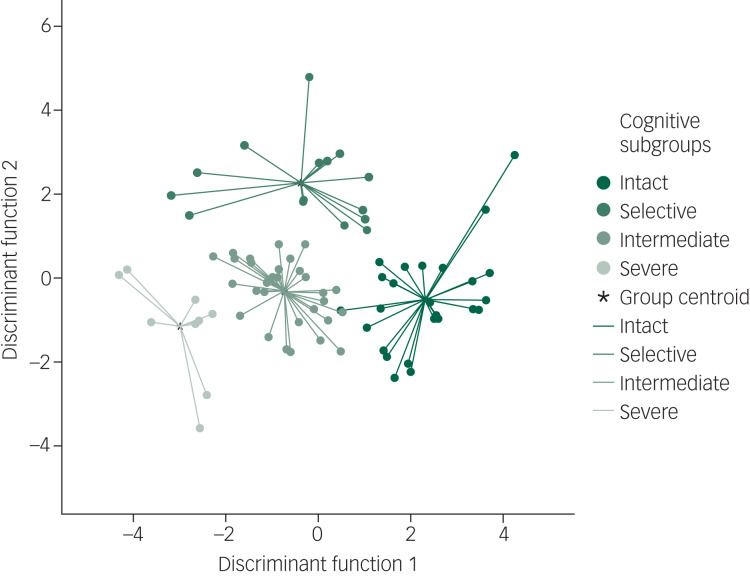
Graphical agglomeration of the cognitive subgroups (cognitively intact; selective deficit in verbal learning and memory; intermediate impairment across all domains; and severe global impairments). Data plots represent participant scattering and clustering based on the discriminant function values. Group centroids represent mean scores for each cluster.

The DFA revealed the presence of two significant discriminant functions, explaining 70% and 29% of the cluster membership variance respectively (Wilks’ *λ* = 0.11, *χ*^2^ = 170.5, *P* < 0.001 and Wilks’ *λ* = 0.43, *χ*^2^ = 63.2 *P* < 0.001 respectively). Verbal learning and memory were the main contributors to participant classification in function 1 (*β* = 0.7), followed by working memory in function 2 (*β* = 0.5). According to the DFA, 97.5% of the original grouped participants were correctly classified, suggesting a valid clustering of the sample. A scatterplot of participants per cluster is shown in Fig. 2.

### Subgroup cognitive profiles

Comparisons between subgroups on cognitive measures are presented in Table 2. As anticipated, the MANCOVA revealed a significant main effect of cluster on all clustering domains (all *P* < 0.001). *Post hoc* pairwise comparisons corrected for multiple testing (5% FDR) showed that participants in the severely globally impaired subgroup performed significantly lower in all clustering domains and the composite cognitive score compared with the cognitively intact subgroup (*d* = 2.4–4.1) and compared with the selectively impaired subgroup with the exception of verbal memory (*d* = 1.7–3.6). Smaller but still largely significant differences were found between participants with intermediate and severe global impairments in all domains but executive functioning (*d* = 1.3–3.1).

**Table 2:** Comparison between subgroups on cognitive measures^a^

		Mean (s.d.)				Main effect statistics			
Domains	Tests	Cognitively intact (*n* = 25)	Selective impairment (*n* = 15)	Intermediate impairment (*n* = 30)	Severe impairment (*n* = 10)	*F*	d.f.	*P*	LSD *post hoc* comparisons
Clustering measures									
Processing speed	Coding; Symbol search	0.29 (0.69)	0.38 (0.49)	−0.42 (0.33)	−1.15 (0.36)	20.671	4,79	<0.001	Int > IM/SG (*d* = 1.3/2.3) Sel > IM/SG (*d* = 2.0/3.4) IM > SG (*d* = 2.1)
Working memory	Digit Span (forward, backward and sequencing)	0.34 (0.44)	0.36 (0.47)	−0.33 (0.23)	−1.05 (0.25)	35.345	4,79	<0.001	Int > IM/SG (*d* = 1.9/3.4) Sel > IM/SG (*d* = 2.1/3.5) IM > SG (*d* = 3.1)
Verbal learning and memory	Verbal Paired Associates I and II	1.01 (0.45)	−1.16 (0.80)	−0.52 (0.54)	−1.32 (0.66)	47.154	4,79	<0.001	Int > all (*d* = 3.0–4.1) IM > SG (*d* = 1.3)
Executive functioning	Hotel test; verbal fluency test (FAS); Matrix Reasoning	0.21 (0.53)	−0.02 (0.56)	−39 (0.55)	−1.03 (0.47)	10.876	4,79	<0.001	Int > IM/SG (*d* = 1.0/2.3) Sel > SG (*d* = 1.9) IM > SG (*d* = 1.2)
Non-clustering measures									
Cognitive screening	MoCA	26.8 (2.2)	25.1 (2.3)	25.9 (1.8)	21.8 (2.5)	11.975	4,79	<0.001	Int > SG (*d* = 2.2) Sel > SG (*d* = 1.3) IM > SG (*d* = 1.9)
Clinician-rated cognitive functioning	FAST cognitive domain	4.2 (2.6)	6.2 (2.2)	6.3 (2.4)	7.3 (1.6)	4.167	4,79	0.004	Int > IM/SG (*d* = 0.8/1.3)
Cognitive decline and cognitive reserve									
Estimated cognitive decline	–	−0.23 (0.37)	−0.89 (0.31)	−0.97 (0.52)	−1.4 (0.44)	15.236	4,79	<0.001	Int > all (*d* = 1.8–2.9) Sel > SG (*d* = 1.3)
Cognitive reserve	TOPF; Vocabulary; years of education	0.37 (0.94)	0.17 (0.78)	−0.21 (0.86)	−0.87 (1.2)	4.628	4,79	0.002	Int > IM/SG (*d* = 0.6/1.2) Sel > SG (*d* = 1.0)

LSD, least significant difference; Int, cognitively intact subgroup; IM, intermediate impairment subgroup; Sel, selective impairment subgroup; SG, severe global impairment subgroup; MoCA, Montreal Cognitive Assessment; FAST, Functional Assessment Short Test; TOPF, Test of Premorbid Functioning.

a. Means and standard deviations for all cognitive measures represent *z*-scores standardised to the normative data of each test. Age was included as a covariate in all analyses. Only *post hoc* comparisons significant at *P* > 0.05 after false discovery rate (5%) correction are reported. Cohen's *d* effect size corrected for unequal subgroup sample sizes (Hedges’ *g*).

### Internally validating cognitive subgroups

The two non-clustering cognitive measures were significantly correlated with the four clustering domains across the sample. The MoCA showed moderate correlations with working memory (*r* = 0.29; *P* = 0.009) and processing speed (*r* = 0.37; *P* = 0.001) and moderate to large correlations with executive functioning (*r* = 0.42; *P* < 0.001) and verbal memory (*r* = 0.47; *P* < 0.001). The cognitive subscale of the FAST was moderately correlated with all domains (*r* = 0.27–0.37; all *P* < 0.01) but processing speed. The severe global impairment subgroup performed significantly worse than all other groups on the MoCA (*d* = 1.3–2.2), while both the intermediate impairment (*d* = 0.8) and the severe global impairment (*d* = 1.3) subgroups had poorer FAST scores than the intact subgroup.

### Cognitive decline and cognitive reserve

**Fig. 3:**
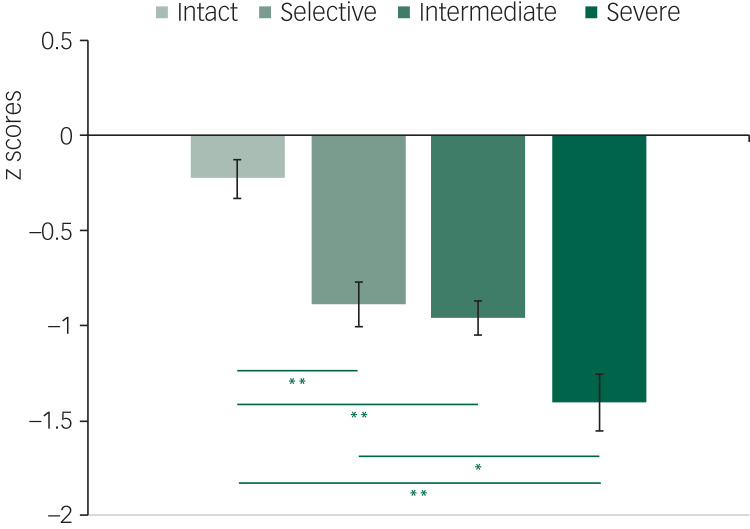
Estimated postmorbid cognitive decline per subgroup (cognitively intact; selective deficit in verbal learning and memory; intermediate impairment across all domains; and severe global impairments), calculated as the discrepancy between current global cognition composite score and premorbid cognitive functioning (Test of Premorbid Function) score. Significant differences (5% false discovery rate correction): *P < 0.1, **P < 0.001.

The MANCOVA for the estimated cognitive decline and the cognitive reserve score revealed significant differences between subgroups for both measures (all *P* < 0.01). Details are reported in Table 2. The discrepancy between the current global cognition composite and the estimated premorbid cognitive functioning (TOPF score) indicated a decline for all subgroups, which gradually increased in magnitude from the cognitively intact to the severe impairment subgroup (Fig. 3). Compared with the intact participants, all other subgroups presented with significantly greater cognitive decline (*d* = 1.8–2.9). This difference was also significant between the selective impairment and the severe impairment subgroups (*d* = 1.3).

As hypothesised, the cognitive reserve score was not correlated with age (*r* = −0.09; *P* > 0.1), age at illness onset (*r* = −0.15; *P* > 0.1), illness duration (*r* = 0.08; *P* > 0.1) and number of mood episodes (*r* = −0.18; *P* > 0.1). The cognitively intact subgroup had a higher cognitive reserve compared with the intermediate impairment (*d* = 0.6) and the severe impairment (*d* = 1.2) subgroups, while a significant difference was found between the selective impairment and the severe impairment subgroups (*d* = 1). Cognitive reserve showed a small, non-significant correlation with estimated cognitive decline across the sample (*r* = −0.2; *P* = 0.08). However, examining this association within each group, cognitive reserve and cognitive decline were strongly correlated for the intact, the intermediate impairment and the severe impairment subgroups (*r* = −0.65 to −0.74; all *P* < 0.05), while the correlation was not significant for the selective impairment subgroup (*r* = 0.05).

### Subgroup clinical characteristics

Table 3 reports the ANOVA results of cluster comparisons for demographic and clinical characteristics. Significant main effects of cluster were found for age, age at diagnosis, the number of psychological therapies previously attended and medication use (*P* = 0.02–0.04). From illness-history variables, only age at onset differed between subgroups, where the selectively and the intermediately impaired participants were diagnosed at an older age compared with the cognitively intact subgroup (*d* = 1 and *d* = 0.8 respectively). Intact participants had undertaken more psychological therapies than severely impaired participants (*d* = 1) and were taking fewer medications compared with the selectively and intermediately impaired subgroups (*d* = 0.8). A significantly smaller proportion of this subgroup used antipsychotics compared with all other subgroups (*d* = 0.7). No differences among subgroups were detected for any other clinical variables or mood measures.

**Table 3:** Comparison between subgroups on demographic, clinical and illness-history variables

Characteristics	Subgroup				Main effect statistics			
	Cognitively intact (*n* = 25)	Selective impairment (*n* = 15)	Intermediate impairment (*n* = 30)	Severe impairment (*n* = 10)	*F* or *χ*^2^	d.f.	*P*	Games–Howell *post hoc* comparisons^a^
Demographic								
Age, years: mean (s.d.)	36.3 (11.8)	45.8 (11.1)	44.2 (13.4)	44.7 (12.2)	2.850	3,79	**0.043**	–
Gender, female/male: %	76/24	53/47	77/23	70/30	3.063	3	0.382	–
Clinical and illness history								
Bipolar disorder type, I/II: %	58/42	60/40	67/33	70/30	1.362	3	0.748	–
History of psychosis, *n* (%)	14 (56)	10 (67)	20 (67)	8 (80)	1.934	3	0.586	–
Age diagnosed, years: mean (s.d.)	25.2 (8.5)	35.6 (12.4)	33.6 (12.8)	30.30 (9.7)	3.638	3,79	**0.016**	Int < Sel/IM (*d* = 1.0/0.8)
Diagnosis duration, years: mean (s.d.)	11.1 (8)	10.2 (9.1)	10.6 (9.7)	14.4 (8.6)	.589	3,79	0.589	–
Symptom duration, years: mean (s.d.)	17.8 (11.6)	22.8 (11.8)	22.3 (14.5)	26.5 (9.4)	1.303	3,79	0.280	–
Untreated illness, years: mean (s.d.)	6.8 (8.2)	12.7 (9.1)	11.7 (10.4)	12.1 (10.7)	1.598	3,79	0.197	–
Number of depressive episodes, mean (s.d.)	10.5 (10.4)	10.9 (8.9)	15.9 (19.7)	8.6 (6.8)	1.026	3,79	0.386	–
Number of (hypo)manic episodes, mean (s.d.)	6.9 (7.1)	8.5 (5.5)	9.3 (8.3)	10.3 (6.1)	.737	3,79	0.533	–
Number of hospital admissions, mean (s.d.)	1.3 (1.3)	2.8 (3.1)	2.7 (2.9)	3.8 (4.3)	2.254	3,79	0.089	–
Current euthymia, months: mean (s.d.)	18.9 (35.5)	16.9 (25.5)	8.4 (12.1)	12.6 (16.3)	0.943	3,79	0.424	–
HRSD-17 score, mean (s.d.)	3.3 (2.3)	3.4 (2.5)	3.3 (2.1)	3.4 (2.1)	0.012	3,79	0.998	–
YMRS score, mean (s.d.)	2 (2.1)	1.7 (2.1)	1.9 (2.1)	2.8 (2.3)	0.569	3,79	0.637	–
Medication and service use								
Lithium, *n* (%)	5 (20)	7 (47)	10 (33)	4 (40)	3.419	3	0.331	–
Anticonvulsants, *n* (%)	14 (56)	9 (60)	20 (67)	4 (40)	2.314	3	0.510	–
Antipsychotics, *n* (%)	13 (52)	13 (87)	24 (80)	9 (90)	9.371	3	**0.025**	Int < all groups (*w* = 0.7)
Antidepressants, *n* (%)	9 (36)	9 (60)	18 (60)	3 (30)	5.313	3	0.150	–
Anxiolytics, *n* (%)	2 (8)	3 (20)	6 (20)	2 (20)	1.819	3	0.611	–
Number of medications (current), mean (s.d.)	1.7 (1.2)	2.8 (1.9)	2.8 (1.6)	2.4 (.69)	3.211	3,79	**0.028**	Int < Sel/IM (*d* = 0.7/0.8)
Number of psychological therapies undertaken, mean (s.d.)	2.6 (1.6)	1.9 (1.1)	1.8 (1.7)	1 (.82)	2.838	3,79	**0.044**	Int > SG (*d* = 1.1)

Int, cognitively intact subgroup; IM, intermediate impairment subgroup; Sel, selective impairment subgroup; SG, severe global impairment subgroup; HRSD-17, 17-item Hamilton Rating Scale for Depression; YMRS, Young Mania Rating Scale.

*P* values in bold indicate significance at *P* < 0.05.

a. Cohen's *d* effect size corrected for unequal subgroup sample sizes (Hedges’ *g*). For categorical variables, Cohen's *w* was computed based on the *χ*^2^ statistic and reflects the mean difference between one subgroup and all the others.

## Discussion

Using an extensive cognitive battery and hierarchical cluster analysis, this study examined the presence of discrete cognitive profiles in a cohort of euthymic people with bipolar disorder to evaluate a clustering solution independent of mood symptoms. We examined the characteristics of these clusters, specifically related to cognitive reserve. Approximately one-third of participants appeared cognitively intact (31%) and another third demonstrated intermediate deficits across all domains (37.5%), with the remainder presenting a selective impairment in verbal learning and memory (19%) or showing severe deficits across all domains (12.5%). The most discriminating cognitive domains for the profiles were verbal learning and memory, followed by working memory and processing speed. Cognitively impaired subgroups presented with greater cognitive decline and poorer cognitive reserve, but few differences were detected in clinical characteristics.

### How do the identified cognitive profiles relate to previous findings?

The four clusters found in this study are in line with previous work on euthymic individuals with bipolar disorder,^18,19^ as well as cross-diagnostic studies including participants from the wider schizophrenia–bipolar disorder spectrum.^9^ The proportion of cognitively intact participants (31%) is very similar to reports in other studies (20–45%),^7^ and above average performance in verbal learning and memory in this subgroup has also been reported.^19^ A relatively small proportion (12.5%) presented with severe deficits across all domains, previously reported to comprise 10–35% of people with bipolar disorder.^7^ A possible explanation for the proportion being at the lower end of this range is that our sample included only individuals in full remission, with higher overall premorbid cognitive functioning and more years in education than previous studies, which might indicate a high-performing sample compared with other cohorts (supplementary Table 1, available at https://doi.org/10.1192/bjo.2020.111). The other two clusters included participants who either experienced a significant impairment in a single domain but otherwise remained intact (19%), or participants with intermediate impairment across all domains (37.5%). These clusters correspond to subgroups described as having ‘selective’ or ‘moderate’ impairment in previous studies reporting four clusters (15–40%).^7^

The division of moderately impaired participants into two distinct clusters was the main difference from previous studies reporting three-cluster solutions. One factor affecting potential clustering is the definition of euthymia used for inclusion. In this study we included only individuals in full remission and previous studies with similar definitions also found four-cluster solutions, with subgroups impaired in a single domain or an intermediate impairment across all domains.^18,19^ In contrast, studies with less restrained definitions or including participants in partial remission have mostly reported three-cluster solutions.^11,35,36^ (supplementary Table 1). Although there is no clear explanation for this disparity, it is possible that reduced cognitive performance in otherwise intact domains due to residual depressive symptoms leads to a shared cluster between participants with selective and intermediate deficits.

The performance of each subgroup across the clustering domains suggested largely distinct cognitive profiles, apart from the cognitively intact and the selective impairment subgroups, where the difference was significant only for verbal memory. This distinction was partially validated by the non-clustering measures. Although the clustering and non-clustering cognitive measures showed small to moderate correlations between subgroups, significant subgroup differences validating the identified profiles for this sample were only found between the intact and the severely impaired participants. These two subgroups were clearly separated on both non-clustering measures, whereas differences observed between the selective impairment and intermediate impairment subgroups were minimal.

Despite multiple studies supporting the existence of distinct cognitive profiles among people with bipolar disorder, these results were solely based on behavioural measures analysis, while potential biological underpinnings of cognitive variability remain largely unknown. Recent neuroimaging findings have pointed to weaker interregional connectivity as a neural mechanism possibly underlying cognitive heterogeneity in subgroups of people with bipolar I disorder clustered on the basis of their performance on executive functioning tasks.^37^ Although this study differed from typical cluster-analytic designs in terms of grouping participants according to their response pattern (i.e. encompassing strengths and deficits in their performance) rather than their normative cognitive performance, it did provide initial evidence on the neurological background of distinct cognitive profiles in bipolar disorder.

### What is the role of cognitive reserve?

Reduced premorbid IQ has been associated with the severe impairment subgroup in previous bipolar disorder and cross-diagnostic cluster-analytic studies.^10,11^ Our study cultivates these findings and indicates a linear pattern of increasingly reduced cognitive reserve across subgroups, gradually reducing from the intact to the severe global impairment subgroup. The reverse pattern was observed for the estimated cognitive decline, and the association between cognitive reserve and cognitive decline was significant for all participants but the selectively impaired ones. On the basis of these findings, it is plausible that differential cognitive performance across subgroups is a function of cognitive reserve for the intact, the intermediate impairment and the severe impairment subgroups. This is consistent with the theoretical concept of cognitive reserve in dementia and schizophrenia spectrum or mood disorders, as a protective mechanism against cognitive decline caused by neuropathology itself^13,38^ or against the adverse cognitive effects of certain treatments (e.g. memory performance after electroconvulsive therapy).^39^

Although cognitive decline after symptom onset was evident across all subgroups, the severely impaired subgroup showed the greatest decline and had the poorest cognitive reserve. Cognitive reserve potentially modifies cognitive performance and explains individual differences in the cognitive course of people with bipolar disorder, with worse outcomes for those with poorer reserve.^17,40^ Recent evidence points to a subgroup of people with bipolar disorder sharing genetic and symptomatic characteristics with schizophrenia.^41,42^ This subgroup presents with a cognitive profile comparable to that of people with schizophrenia, including a neurodevelopmental trajectory characterised by poor cognitive reserve.^43,44^ This neurodevelopmental hypothesis is further supported by evidence for the existence of discrete cognitive clusters in young offspring of people with bipolar disorder prior to illness onset.^45^

In our sample, participants with severe deficits across domains potentially represent this subgroup of people with bipolar disorder following a cognitive course defined by reduced cognitive reserve. This argument is strengthened by non-significant or inconsistent findings for subgroup differences in clinical and illness-history characteristics (e.g. diagnostic subtype, illness duration, number of episodes), suggesting that cognitive variability in people with bipolar disorder cannot be entirely attributed to illness progression. Pending confirmation by longitudinal studies, cognitive reserve may be a factor driving disparate illness trajectories and a characteristic that may predict the extent of cognitive impairment in people with bipolar disorder.

### Do clusters differ in their clinical characteristics?

No significant differences were observed between subgroups in demographic characteristics and most illness-history variables, which suggests that subgroup allocation and cognitive variability in our sample was largely independent of clinical characteristics. Likewise, subthreshold depressive and (hypo)manic symptoms were balanced between subgroups, which has been previously reported for cluster-analytic studies in bipolar disorder.^7^

Although the duration of illness and the number of mood episodes did not differ between subgroups, cognitively intact participants were diagnosed at a younger age than participants in the selective impairment and intermediate impairment subgroups. People with bipolar disorder experience significant delays in receiving the correct diagnosis and in receiving treatment.^46^ As a result, differences in age at diagnosis may not reflect differences in age at symptom onset. To examine that further, we computed the duration of untreated illness (DUI), defined as the discrepancy between the time when they first experienced symptoms and when they received their diagnosis, and compared it across subgroups. Although cognitively intact participants received their diagnosis 7 years on average after symptom onset and DUI exceeded 10 years for all other subgroups, this difference was not statistically significant. Thus, our findings do not support that greater DUI is associated with more severe illness course in terms of cognitive impairments.^47^

Subgroup differences were evident in treatment characteristics. To the best of our knowledge, this is the first study reporting that cognitively intact participants had undertaken more psychological therapies than those with severe impairment, but it is unclear whether this reflects a neuroprotective effect of psychological therapies or if it simply reflects the capacity of participants in this subgroup for better treatment access and adherence. A smaller proportion of intact participants were using antipsychotics compared with the other subgroups. This was despite the lack of any significant subgroup differences in history of psychosis and has been previously reported in cluster-analytic studies.^18^ The relationship between treatment and potential cognitive outcomes is complex, as greater use of antipsychotics may be an indicator of greater illness severity, particularly regarding increased risk for manic relapse. Studies examining the cognitive course of patients who discontinued or reduced their antipsychotic medication may clarify this question.^48^

### Limitations

This was a cross-sectional study so we cannot speculate on the stability of these cognitive subgroups. Likewise, estimated cognitive decline following illness onset was calculated using cross-sectionally collected measures. In addition, the sample size was relatively small, although it did meet the minimum requirements for a cluster analysis. Therefore, findings on the role of cognitive reserve need to be interpreted with caution and require further replication in larger studies. There is a possibility that effect sizes were inflated owing to our sample size, but we did use a conservative approach for multiple comparison correction to reduce false positives. We therefore consider the identified subgroups and the detected differences reasonable. The neuropsychological battery used was less extensive, with fewer tests per domain compared with some of the previous studies, but cognitive scores did not differ significantly within each domain and the variation in participant scores from the domain mean was small, indicating limited estimation bias. Finally, we did not measure psychiatric comorbidities that could potentially affect cognitive performance.

### What are the clinical implications?

We recently reviewed the literature on cognitive remediation therapies targeting cognitive and functional outcomes for people with bipolar disorder.^49^ Findings are promising but still inconsistent across studies. Cognitive heterogeneity in study samples may underly this inconsistency. Individuals with different cognitive profiles can have different outcomes and some adaptations may be necessary in treatment provision. Different patient clusters may require modifications to adhere to and benefit from treatment, for example longer or more intensive therapy periods for those more severely impaired.^50^ Our findings also suggest that considering the concept of cognitive reserve might be relevant in the context of designing and delivering cognitive interventions, either as a potential treatment target or as a factor enabling treatment engagement and outcomes. Clinical services for people with bipolar disorder should introduce cognitive assessment into their screening process for new or recurring patients. The cognitive domain mostly contributing to differentiation of the four cognitive profiles was verbal learning and memory, and previous research has associated verbal memory performance with future functional outcomes.^51^ Hence, it might be a suitable domain for clinical services to screen in order to quickly and effectively differentiate between cognitively intact and compromised patients.

## Data Availability

The anonymised data-set is available from the corresponding author on request.

## Supplementary material

For supplementary material accompanying this paper visit http://dx.doi.org/10.1192/bjo.2020.111. click here to view supplementary material
