# Supplementary material for: Role of cognitive reserve in cognitive variability in euthymic individuals with bipolar disorder: cross-sectional cluster analysis
Source: BJPsych Open. 2020 Oct 30;6(6):e133. doi: 10.1192/bjo.2020.111 (PMC7745228; doi:10.1192/bjo.2020.111)
Supplement: Supplementary file 1 [file bjosup.zip › S2056472420001118sup001.docx]

**Supplementary table 1.** Potential sources of heterogeneity in cluster-analytic studies for people with bipolar disorder.

| **Studies** | **N** | **Age (y)** | **Education (y) / Premorbid IQ** | **Mood**  **symptoms** | **BD type 1 (%) /  Age of diagnosis (y) / Illness duration (y)** | **Cognitive  domains** | **Clustering method** | **Clusters reported** | **Cluster validation  (% correctly classified)** |
| --- | --- | --- | --- | --- | --- | --- | --- | --- | --- |
| Bora et al., 2016 | 556 | 36 | 11 / - | Euthymic | 92 / - / 12.5 | ExF | LCA | 4 | - |
| Burdick et al., 2014 | 136 | 41 | 14 / 98 | Partially  remitted | 77 / 20.6 / - | Att, PrSp, WM, VisLM, VerLM, ExF, SoCog | HCA | 3 | DFA (-)  Split-half sample |
| Jensen et al., 2016 | 193 | 36 | 15 / - | Euthymic partially  remitted | 58 / 22 / 15 | PrSp, WM, VerLM, ExF | HCA | 3 | DFA (90%) |
| Jimenez et al., 2017 | 113 | 48 | 14 / 109 | Partially  remitted | 72 / 26.5 / 22 | Att, PrSp, WM, VerLM, ExF, SoCog | HCA | 3 | DFA (88.5%) |
| Lima et al., 2019 | 73 | 49 | 11 / - | Euthymic | 89 / 33.5 / 15.5 | Att, PrSp, WM, VerLM, ExF, SoCog | HCA | 3 | DFA (90%) |
| Roux et al., 2017 | 258 | 41 | 14 / - | Euthymic | 55.5 / 25 / - | Att, PrSp, WM, VerLM, ExF | HCA | 4 | DFA (94%) |
| Russo et al., 2017 | 60 | 38 | - / 98 | Partially  remitted | 87 / - / - | Att, PrSp, VerLM, ExF | HCA | 3 | DFA (100%) |
| Sole et al., 2016 | 51 | 33 | 14 / 107 | Euthymic | 0 / 25 / 17.5 | Att, PrSp, WM, VisLM, VerLM, ExF | HCA | 3 | DFA (90%) |
| *Notes:* Att: Attention; ExF: Executive functioning; DFA: Discriminant function analysis; HCA: Hierarchical cluster analysis; LCA: Latent class analysis; PrSp: Processing speed; SoCog: Social cognition; VerLM: Verbal learning and memory; VisLM: Visual learning and memory; WM: Working memory. | | | | | | | | | |
